# Bioactive Oleanane, Lupane and Ursane Triterpene Acid Derivatives

**DOI:** 10.3390/molecules171012197

**Published:** 2012-10-17

**Authors:** Maria de L. e Silva, Juceni P. David, Lidércia C. R. C. Silva, Rauldenis A. F. Santos, Jorge M. David, Luciano S. Lima, Pedro S. Reis, Renato Fontana

**Affiliations:** 1 Faculdade de Farmácia, Universidade Federal da Bahia, Rua Barão de Geremoabo, s/n, 41810-290, Salvador, BA, Brazil; Email: lurdinhafarma@yahoo.com.br (M.L.S.); juceni@ufba.br (J.P.D.); liu@ufba.br (L.C.R.C.S.); 2 Instituto de Quimica, Universidade Federal da Bahia, Rua Barão de Geremoabo, s/n, 41810-290, Salvador, BA, Brazil; Email: rauldenis_fonseca@yahoo.com.br; 3 Instituto Federal da Bahia, Campus Porto Seguro, Br 367, km 57.5 Fontana I - Porto Seguro, 45810-000, BA, Brazil; Email: lucianolim@yahoo.com.br; 4 Núcleo de Pesquisa em Biodiversidade e Biotecnologia – Biotec, Universidade Federal do Piauí, Campus de Parnaíba. 64202-020, Parnaíba, PI, Brazil; Email: reisps@ufpi.edu.br; 5 Departamento de Ciências Biológicas, Universidade Estadual de Santa Cruz, Km 16 Rodovia Ilhéus-Itabuna 45662-000, Ilhéus, BA, Brazil; Email: rfontana@uesc.br

**Keywords:** *Eriope blanchetii*, Lamiaceae, triterpene derivatives, *Artemia salina* lethality, antimicrobial activity

## Abstract

Betulinic, ursolic and oleanolic acids isolated from the aerial parts of *Eriope blanchetii* (Lamiaceae) were subjected to different esterification reactions, yielding 12 C-3 position ester derivatives. All compounds were identified using spectroscopic techniques, such as IR, ^1^H-NMR and MS. The derivatives were further investigated for their antioxidant level, *Artemia salina* lethality and antimicrobial activity.

## 1. Introduction

Triterpenes are a class of natural products present in all organisms, especially in plants. The triterpene acids exhibit unique and important biological and pharmacological activities, including anti-inflammatory, antimicrobial, antiviral, cytotoxic and cardiovascular effects [1,2,3,4,5]. Synthesis of triterpene derivatives is a strategy to obtain compounds with enhanced bioactivity by, for example, the introduction of electron-withdrawing/donating groups [6].

Betulinic, ursolic and oleanolic acids are the main triterpenes present in *Eriope blanchetii*, a shrub belonging to the Lamiaceae family. It is an endemic Brazilian plant and occurs at the sandy soils of the Bahia coast [7]. To date, there are few chemical studies involving this species. Triterpenes and bioactive lignans have been isolated from its organic extracts [8,9].

Previous studies have demonstrated that triterpene acids esterified with cinnamic acid derivatives presented activity against *Mycobacterium tuberculosis* [10]. The phthalic ester of betulinic acid was shown to be cytotoxic [11], and the dimethylsuccinic ester of betulinic acid was active against human immunodeficiency virus (HIV) [12]. In addition, a number of other ester derivatives of ursolic and oleanolic acids have anti-inflammatory activities [13]. This work describes the synthesis of ester derivatives of betulinic, ursolic and oleanolic acids and the determination of the cytotoxicity of these compounds as determined by their lethality towards *Artemia salina* (brine shrimp test) and their antimicrobial activity against *Escherichia coli* (ATCC 25922) and *Staphylococcus aureus* (ATCC 25923).

## 2. Results and Discussion

*Eriope blanchetii* is an endemic Brazilian plant that produces considerable amounts of betulinic acid, as well as oleanolic and ursolic acids. These compounds were re-isolated from the aerial parts of this plant using chromatographic techniques. Structural identification of these acids was based on a comparison of their ^1^H- and ^13^C-NMR spectra with literature spectra for the corresponding methyl ester derivatives [14].

The three triterpene acids were esterified with different anhydrides and/or acyl chlorides. These procedures yielded twelve triterpene acyl derivatives **1a**–**d**, **2a**–**d**, **3a**–**d**. The compounds **1d**, **2d** and **3d** are new compounds. The structures of these derivatives were easily confirmed by MS, IR and NMR by comparing their respective data with that of the parent betulinic, oleanolic and ursolic acids. The presence of additional and characteristic υ_C=O_ and υ_C–O_ stretches in the IR spectra indicates the presence of an ester functionality. In the ^1^H-NMR spectra of these compounds, the esterified products can be identified by the signal of the deshielded H-3 (δ 3.7–4.7) compared to the free H-3 (δ 3.1–3.4) of the triterpene acids (Table 1).

**Table 1:** H-3 ^1^H-NMR data for betulinic, oleanolic and ursolic acids and their ester derivatives [300 MHz, δ (ppm) *J* (Hz)].

Compound	Solvent	δ H-3
**1**	Py	3.5 ( *t*, 7.0 Hz)
**1a**	Cd	4.48 ( *t*, 7.7 Hz)
**1b**	Cd	4.48 ( *t*, 7.7 Hz)
**1c**	Ac	3.9 ( *t*, 7.7 Hz)
**1d**	Ac	3.7 ( *t*, 7.1 Hz)
**2**	Py	3.5 ( *t*, 8.0 Hz)
**2a**	Cd	4.51 ( *t*, 7.7 Hz)
**2b**	Ac	4.49 ( *m*, 7.7 Hz)
**2c**	Ac	4.7 ( *t*, 7.7 Hz)
**2d**	Ac	4.35 ( *t*, 7.7 Hz)
**3**	Py	3.48 ( *t*, 7.8 Hz)
**3a**	Cd	4.51 ( *dd*, 6.0 and 8.6 Hz)
**3b**	Cd	4.51 ( *dd*, 5.6 e 8.9 Hz)
**3c**	Cd	4.76 ( *dd*, 5.6 e 8.9 Hz)
**3d**	Cd	4.75 ( *t*, 8.9 Hz)

Py = pyridine-*d*_5_, Ac = acetone-*d*_6_, Cd = CDCl_3_.

**Table 2:** Cytotoxicity evaluation by the brine shrimp test of triterpene acid derivatives.

Compound	Lethality towards *Artemia salina*	
	CL_50_ (µg/mL)	SD
**1a**	>1,000 μg/mL	-
**1b**	>1,000 μg/mL	-
**1c**	>1000 μg/mL	-
**1d**	117.1 μg/mL	0.418
**2a**	>1,000 μg/mL	
**2b**	>1,000 μg/mL	-
**2c**	>1,000 μg/mL	-
**2d**	477.2 μg/mL	0.304
**3a**	>1,000 μg/mL	-
**3b**	>1,000 μg/mL	-
**3c**	>1,000 μg/mL	-
**3d**	>1,000 μg/mL	-

SD with 95% confidence interval (µg/mL).

The brine shrimp test was then used to determine the CL_50_ of each of the prepared compounds. Compounds with a CL_50_ greater than 1,000 µg/mL can be considered inactive, those with a CL_50_ less than 100 µg/mL are very active, and those with a CL_50_ between 100 and 900 µg/mL are considered moderate [15]. Table 2 shows the results observed for the derivatives. Most of the prepared compounds are inactive, but 3β-(3-chlorobenzoyl) betulinic acid (**1d**) showed a remarkable activity (CL_50_ = 117.1 μg/mL). Betulinic acid is known for its antitumor activities, so this observation can explain the higher activity of **1d** compared to **2d** and **3d**.

Aliphatic compounds with no conjugated double bonds usually do not show considerable antioxidant activities [16], but triterpenes esterified with cinnamic acid derivatives have been known to exhibit scavenging activity when employing the DPPH reagent [17]. All of our derivatives were subjected to this test, and as expected, the majority of the compounds were unable to quench the free radical (Table 3), but 3β-(3-chlorobenzoyl) betulinic acid (**1d**) had an IC_50_ similar to quercetin, the positive control employed. Betulinic, ursolic and oleanolic acids are also known to possess antimicrobial activities. The derivatives were inactive at 80 and 240 µg doses when tested against *E. coli* and *S. aureus*. Therefore, the presence of a free hydroxyl group at C-3 appears to be important for activity.

**Table 3:** Scavenging activity observed for **1d**, **2a** and **2d** in the DPPH test.

Compound	IC_50_ ± RSD (μg/mL)
**2d**	1444 ± 2.0
**1d**	23.41 ± 0.9
**2a**	44.58 ± 0.7
**Quercetin (positive control)**	23.18 ± 1.4

## 3. Experimental

### 3.1. General Procedures

^1^H-NMR (300 MHz) experiments were carried out on a Varian Gemini 2000 or a Bruker AMX300; chemical shifts were recorded in δ (ppm) from the solvent peak relative to TMS. APCI and ESI-MS data were obtained on a Shimadzu LCMS-2010, and IR spectra were recorded on a Varian 640-IR spectrophotometer. Column chromatography was carried using silica gel 60 (Akros 0.04–0.073 mm), and silica gel TLC plates employing iodine fumes, the Libermann-Burchard spray reagent, and UV light (254/366 nm) were used to monitor chromatographic purification.

### 3.2. Plant Material

Botanical material of *Eriope blanchetii* (Lamiaceae) was collected in May 2008 at Parque Metropolitano do Abaeté, Salvador, Bahia State, a region where “restinga” vegetation is prevalent. The species was identified by Prof. Maria L. S. Guedes, and a voucher is deposited at Herbário Prof. Alexandre Leal Costa, Instituto de Biologia da UFBA, under number 045599.

### 3.3. Extraction and Isolation

The dried and powdered aerial parts of *E. blanchetii* (1.7 kg) were repeatedly extracted with MeOH (4 L, 48 h) at room temperature, and the crude extract was immediately partitioned. Firstly it was solubilized with MeOH–H_2_O (9:1) and extracted by hexane and sequentially the hydroalcoholic portion was solubilized with MeOH–H_2_O (6:4) and then partitioned employing CHCl_3_. After the evaporation of the CHCl_3_ under vacuum, the extract obtained (47.2 g) was loaded onto the silica gel column and eluted with a CHCl_3_:MeOH gradient (95:5 ➔ 3:2). The fractions rich in triterpenes were identified by TLC using the Liebermann-Burchard spray reagent. These fractions were then loaded onto a Sephadex LH-20 column and eluted with CHCl_3_–MeOH (2:3); the eluate was then loaded onto a silica gel column and eluted with mixtures of hexane/EtOAc. These procedures yielded pure betulinic (2.5 g), oleanolic (903 mg) and ursolic (570 mg) acids.

### 3.4. Synthesis of the Ester Derivatives

**Scheme 1:**
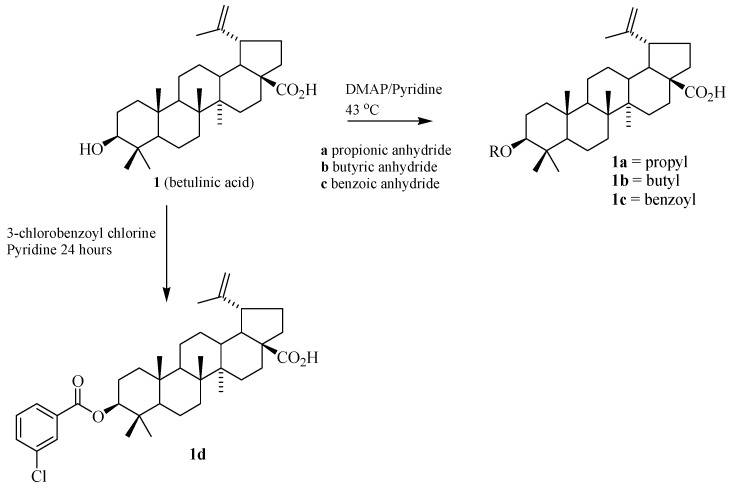
Preparation of the ester derivatives from betulinic acid.

**Scheme 2:**
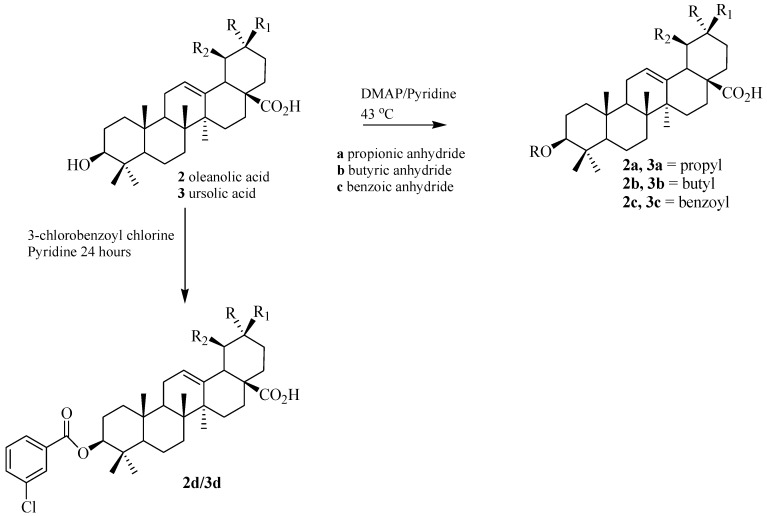
Preparation of the ester derivatives from oleanolic and ursolic acids.

Ester derivatives of the triterpene acids were synthesized via reaction with anhydrides. The triterpene acids (20 mg), pyridine (2 mL) and 2,4-dimethylaminepiridine (DMAP, 2.0 mg) were placed in a 50 mL flask. Sequentially, propionic or butyric anhydride (0.4 mL) or benzoic anhydride (10 mg) were added to each flask. The mixtures were stirred for 24 h at 45 °C. The solvent was removed. The products were then dissolved in CHCl_3_ and treated with H_2_O, dried with Na_2_SO_4_, filtered and the solvent removed twice under vacuum. The products were purified by silica gel column chromatography, with hexane–EtOAc (9:1) as the eluent. Table 4 gives the yields from this procedure for the triterpene acid esters (Scheme 1 and Scheme 2).

**Table 4:** Yields* of purified ester triterpene acid derivatives.

Triterpene acid derivatives	m (mg)	Yield (%)
**1a**	1.7	10
**1b**	1.7	10
**1c**	13.5	56
**1d**	8.1	33
**2a**	1.4	9
**2b**	1.3	7
**2c**	8.1	33
**2d**	17.1	66
**3a**	12.7	55
**3b**	13.8	60
**3c**	7.3	37
**3d**	19.1	69

* The yields are expressed as purified compound. In some of the reactions the subproducts obtained from the anhydrides and acid chlorides presented similar Rf in chromatography and the ester purification was not complete.

Ester derivatives of the triterpene acids were also prepared with 3-chlorobenzoyl chloride. The three triterpene acids (20 mg) and pyridine (2 mL) were separately placed in a 50 mL flask. Next 3-chlorobenzoyl chloride (23 µL) was added, and the mixtures were stirred in an ice bath for 24 h. The solvent was then removed, and the products were purified by silica gel column chromatography, with hexane–EtOAc (8:2) as the eluent. Table 4 gives the yields from this procedure for the esters of the triterpene acids (Scheme 1 and Scheme 2).

*3β-Propanoyl betulinic acid* (**1a**) APCI-MS *m/z* 511 [M–H]; ^1^H-NMR (CDCl_3_), δ ppm: 5.1 and 5.0 (1H, s, H-29); 4.48 (1H, *J* = 7.7 Hz, t, H-3); 2,02 (1H, *J* = 11 Hz, d, H-18).

*3β-Butanoyl betulinic acid* (**1b**) APCI-MS *m/z* 525 [M–H]; ^1^H-NMR (CDCl_3_), δ ppm: 5.1 and 5.0 (2H, s, H-29); 4,48 (1H, *J* = 7.7 Hz, t, H-3); 2.02 (1H, *J* = 11 Hz, d, H-18).

*3β-Benzoyl betulinic acid* (**1c**). APCI-MS *m/z* 559 [M–H]; ^1^H-NMR (CDCl_3_), δ ppm: 8,15 (2H, *J* = 1,7 and 8.3 Hz, dt, H-3', 7'); 7,65 (1H, *J* = 1.3 and 7.5 Hz, tt, H-5'); 7.5 (2H, *J* = 1.5 and 8 Hz, td, H-4' and 6'); 4.62 and 4.72 (1H, s, H-29); 3.9 (1H, *J* = 7.7 Hz, t, H-3); 2.07 (1H, *J* = 11 Hz, d, H-18).

*3β-3-Chlorobenzoyl betulinic acid* (**1d**). White power. M.p. 165.9–166.4 °C. APCI-MS *m/z* 593 [M–H]; IR (KBr, cm^−1^): 3522–3281 (υ_OH_), 2925–2852 (υ_C–H_), 1689 (υ_C=O_), 1684 (υ_C=O_), 1575 (υ_C=C_), 1303–1263 (υ_C–O_), 847 (υ_C–Cl_). ^1^H-NMR (acetone-*d*_6_), δ ppm: 4.72 and 4.62 (1H, s, H-29); 3.7(1H, *J* = 7.0 Hz, t, H-3); 2.04 (1H, *J* = 11.0 Hz, d, H-18).

*3β-Propanoyl oleanolic acid* (**2a**): APCI-MS *m/z* 511 [M–H]; ^1^H-NMR (CDCl_3_), δ: 5,25 (1H, s, H-12); 4.51 (1H, *J* = 7.7 Hz, t, H-3); 2.34 (2H, *J* = 7.5 Hz, q, H-2'); 2.2 (1H, *J* = 11.0 Hz, d, H-18).

*3β-Butanoyl oleanolic acid* (**2b**): APCI-MS *m/z* 525 [M–H]; ^1^H-NMR (300 MHz, acetone-*d*_6_), δ ppm: 4.49 (1H, *J* = 7,7 Hz, m, H-3).

*3β-Benzoyl oleanolic acid* (**2c**): APCI-MS *m/z* 525 [M–H]; ^1^H-NMR (acetone-*d*_6_), δ ppm: 8.15 (2H, *J* = 1.7 and 8.3 Hz, dt, H-3', 7'); 7.65 (1H, *J* = 1.3 and 7.5 Hz, tt, H-5'); 7.5 (2H, *J* = 1.5 and 8.0 Hz, td, H-4' e 6'); 5.23 (1H, s, H-12); 4.7 (1H, *J* = 7.7 Hz, t, H-3).

*3β-3-Chlorobenzoyl oleanolic acid* (**2d**): White power. M.p. 152.9–154.1 °C APCI-MS *m/z* 593 [M–H]; IR (KBr, cm^−1^): 3511–3312 (υ_OH_), 2925–2852 (υ_C–H_), 1712 and 1690 (υ_C=O_), 1457 (υ_C=C_), 1303–1263 (υ_C–O_), 894 (υ_C–Cl_). ^1^H-NMR (acetone-*d*_6_), δ ppm: 7.99 (1H, s, H-3'); 7.99–7.96 (1H, m, H-7'); 7.6 (1H, d, H-5'); 7.5 (1H, t, H-6'); 4.35 (1H, *J* = 7.7 Hz, t, H-3); 2.3 (1H, *J* = 11 Hz, d, H-18).

*3β-Propanoyl ursolic acid* (**3a**): White power. M.p. 266.5–267.3 °C. IR (KBr, cm^−1^): 3006–2879 (υ_C–H_), 1734 and 1712 (υ_C=O_), 1222 (υ_C–O_). ^1^H-NMR (CDCl_3_), δ ppm: 5.2 (1H, *J* = 3.3 Hz, t, H-12); 4.51 (1H, *J* = 6.0 and 8.6 Hz, dd, H-3); 2.3–2.4 (2H, *J* = 7.5 Hz, q, H-2'); 2.2 (1H, *J* = 11 Hz, d, H-18).

*3β-Butanoyl ursolic acid* (**3b**): White power. M.p. 265.0–265.1 °C. IR (KBr, cm^−1^): 3006–2927 (υ_C–H_), 1734 and 1714 (υ_C=O_), 1222 (υ_C-O_). ^1^H-NMR (CDCl_3_), δ ppm: 5.2 (1H, *J* = 3.3 Hz, t, H-12); 4.51 (1H, *J* = 5.6 and 8.9 Hz, dd, H-3); 2.3–2.4 (2H, *J* = 7.2 Hz, t, H-2'); 2.2 (1H, *J* = 11 Hz, d, H-18).

*3β-Benzoyl ursolic acid* (**3c**): White power. M.p. 197.3–199.5 °C. IR (film, cm^−1^): 3006–2926 (υ_C–H_), 1714 (υ_C=O_), 1222 (υ_C–O_). ^1^H-NMR (CDCl_3_), δ ppm: 8.15 (2H, *J* = 1.7 and 8.3 Hz, dt, H-3', 7'); 7.65 (1H, *J* = 1.3 and 7.5 Hz, tt, H-5'); 7.5 (2H, *J* = 1.5 and 8.0 Hz, td, H-4' and 6'); 5.3 (1H, s, H-12); 4.76 (1H, *J* = 5.6 and 8.9 Hz, dd, H-3); 2.25 (1H, *J* = 11 Hz, d, H-18).

*3β-3-Chlorobenzoyl ursolic acid* (**3d**): White power. M.p. 155.2–157.9 °C. IR (KBr, cm^−1^): 3084–2867 (υ_OH_), 3005–2925 (υ_C–H_), 1721 (υ_C=O_), 1693 (υ_C=O_), 1572 (υ_C=C_), 1291–1256 (υ_C–O_), 846 (υ_C–Cl_). ^1^H-NMR (CDCl_3_), 8.1 (1H, *J* = 1.7 and 2.5 Hz, dd, H-3'); 8.0 (1H, *J* = 1.0 and 7.5 Hz, dt, H-7'); 7.5 (1H, *J* = 1.0 and 8.0 Hz, td, H-5'); 7.4 (1H, *J* = 7.8 Hz, t, H-6'); 5.2 (1H, *J* = 3.3 Hz, t, H-12); 4.75 (1H, *J* = 8.9 Hz, t, H-3); 2.2 (1H, *J* = 11 Hz, d, H-18).

### 3.5. Biological Tests

The brine shrimp lethality test was performed according to Serrano *et al.* [18] with minor modifications [8]. Radical scavenging activities of plant extracts were determined through spectrophotometry using the 1,1-diphenyl-2-picrylhydrazyl (DPPH) scavenging radical assay [19]. The radical scavenging ability was calculated by the formula, % I = [(AbsB − AbsA)/AbsB] × 100, and the IC_50_ was determined from the linear decrease in the inhibition percentage.

All assays were performed in triplicate, and the test results were analyzed using the two-tailed Student's t-test at a significance level of *p* < 0.05. DPPH IC_50_ values with 95% confidence intervals were determined using the regression method with the Analyse-it software. BST LC_50_ values with 95% confidence intervals were determined using the probit analysis method of the Stats Direct statistical software. When required, the results were found by extrapolation of the straight line.

The antimicrobial activity tests on the oils were performed using the method of diffusion in agar previously described in [20]. The oils were tested against gram-positive and gram-negative species, *Staphylococcus aureus* (ATCC 25923) and *Escherichia coli* (ATCC 25922), respectively. A suspension of the tested microorganism (0.5 of MacFarland Scale) was spread onto Petri plates with Mueller Hinton agar (Difco Laboratories, Detroit, MI, USA). Then, 240 and 80 µg of the triterpenes were added to a 2 mm diameter area of the paper dishes. The plates were incubated at 37 °C for 24 h. After this period of time, the diameters of the inhibition zones were measured using a caliper rule and expressed in millimeters.

## 4. Conclusions

The esterification reactions of betulinic, oleanolic and ursolic acids led to preparation of several triterpene C-3 position derivatives. Among these compounds 3β-3-chlorobenzoyl betulinic acid (**1d**), 3β-3-chlorobenzoyl oleanolic acid (**2d**) and 3β-3-chlorobenzoyl ursolic acid (**3d**) are new. Betulinic, ursolic and oleanolic acids are also known to possess antimicrobial activities but the derivatives were inactive when tested against *E. coli* and *S. aureus*, indicating that the presence of a free hydroxyl group at C-3 appears to be important for activity. Nevertheless compound **1d** showed activity in the Brine Shrimp Test and exhibited scavenging activity when employing the DPPH reagent.
